# The incredible complexity of RNA splicing

**DOI:** 10.1186/s13059-016-1121-y

**Published:** 2016-12-30

**Authors:** Christelle Robert, Mick Watson

**Affiliations:** The Roslin Institute and Royal (Dick) School of Veterinary Studies, University of Edinburgh, Edinburgh, UK

## Abstract

Alternative splice isoforms are common and important and have been shown to impact many human diseases. A new study by Nellore et al. offers a comprehensive study of splice junctions in humans by re-analyzing over 21,500 public human RNA sequencing datasets.

## Introduction

A newly published study by Nellore et al. in *Genome Biology* provides us with the most comprehensive view of human transcriptome splicing to date, having (re)analyzed over 21,500 RNA sequencing (RNA-seq) datasets and discovered 56,865 novel splice junctions [1].

RNA splicing is a post-transcriptional RNA processing mechanism occurring in eukaryotic organisms whereby introns are removed from pre-mRNA leading to mature mRNA molecules, or transcripts, consisting of joined exons. The process of RNA splicing generates distinct transcript variants of the same gene, referred to as alternative transcript isoforms, the translation of which leads to distinct protein products. Thus, alternative splicing is a critical process that ensures protein diversity, with most of the multi-exon genes in humans generating multiple alternative transcript isoforms.

## Alternative splicing affects human disease

Dysregulation of alternative splicing can have major functional consequences through the expression of abnormal isoforms that contribute to disease progression. Isoform switching, where the most abundant transcript isoform has changed between two conditions (e.g.*,* cancer and normal cells) is a common mechanism. Recently, Sebestyén et al. [2] reported recurrent isoform switches for known tumor-driver genes (e.g.*, PPARG*, *MITF*, and *MYH11*) across seven cancer types that resulted in altered gene function; and (amongst many others) aberrant splicing mutations have been reported in muscular dystrophy [3] and cystic fibrosis [4].

## RNA-seq as an incredibly powerful method for splice junction discovery

RNA-seq has now become the standard method to analyze the transcriptome, the complete set of transcripts expressed in a given cell. This approach is commonly used to identify the diverse set of transcript types (e.g.*,* mRNA, noncoding RNAs) and their isoform structure (splicing patterns); to quantify transcript-level expression and the changes in expression under various experimental conditions; and to discover novel transcript isoforms or splice junctions; though care must be taken as accurate alignment and quantification is difficult due to the high similarity between some transcripts and genes [5].

Remarkably, Nellore et al. have re-analyzed over 21,500 public RNA-seq datasets, producing the most comprehensive catalogue of splice junctions to date, as well as tracking the annotation of human RNA splicing over time [1].

## Most common junctions are annotated but many rare junctions are not

Nellore et al. find that most of the reads that map to splice junctions map to junctions that are already known; specifically, in 10,090 of 10,311 datasets that met the authors’ filtering criteria, over 95% of junction reads overlap junctions found in the existing annotation. However, although most splice junctions with high read coverage have been documented, there remains a large number of splice junctions that occur across multiple samples that have not. For example, in 3389 samples from the same set (*n* = 10,311), fewer than 80% of the observed junctions are annotated. In total, Nellore et al. report 56,865 novel junctions (18.6%) found in at least 1000 samples. Thus, comparison of multiple independent studies can reveal many unannotated junctions.

## Junction discovery power is influenced by read depth and length

Nellore et al. confirm that variation in unannotated junction expression across samples strongly correlates with both junction sequencing depth and read length. High read coverage across splice junctions provides stronger evidence that it is real and expressed; and an increased read length allows for a larger proportion of reads to be mapped across splice junctions. Thus, both parameters, read depth and read length, strongly influence junction discovery power.

## Most junctions have now been discovered…in human

From 2009 to 2013, splice junction discovery has increased over time with spikes of discovery mostly due to large-scale sequencing projects such as the Human Reference Epigenome Mapping Project [6] (with over 200,000 newly discovered junctions), followed by ENCODE [7] and the Illumina Body Map 2.0 projects. By 2013, the splice junction discovery process reached a plateau, at which point 96.1% of annotated junctions were already discovered. For example, the large-scale GEUVADIS [8] project contributed relatively few novel well-supported splice junctions from lymphoblastoid cell lines, as those cell lines had been well-studied by that time.

## What this means for studies in other species

Accurate gene-level and transcript-level expression analyses often rely on the completeness of transcript and splice junction annotation, and research suffers if that annotation is incomplete. Unfortunately, such information is not at the same level of completion for species other than human—beyond human and mouse, other animal genomes can lack up to 20 megabases of annotation [9]—and even for species as well-studied as human, it is now clear that the transcript annotations are not fully complete.

The effort of Nellore et al. provides an unprecedented insight into the splice junction usage in humans through large-scale RNA-seq data analysis and further highlights the need for similar studies in other less well-characterized species [10]. The data and resource provided by Nellore et al. will be of importance to anyone studying RNA in humans and will specifically impact on our ability to study splice variation effects in human disease.

## Abbreviations

RNA-seq: RNA sequencing
